# Six-Month Survivorship Prediction in Spinal Metastatic Patients by Oncologists Shows Reliable Prognostication

**DOI:** 10.1177/21925682231218712

**Published:** 2023-11-27

**Authors:** Kofi Cox, Hassam Ahmed, Priyanshu Saha, Wing Kin Liu, Katharine Aitken, Jason Bernard, Timothy Bishop, Pawan Minhas, Marios Papadopoulos, Francis Johnston, Alicia Piggott, Erlick Pereira, Darren Lui, Mehran Afshar

**Affiliations:** 1 Department of Medicine, 4915 St. George’s University of London, London, UK; 2 Department of Oncology, 156611 St. George’s University Hospitals NHS Foundation Trust, London, UK; 3 Department of Radiotherapy, 4970 Royal Marsden Hospital, London, UK; 4 Department of Complex Neurosurgery, Atkinson Morley Wing, 156611 St. George’s University Hospitals NHS Foundation Trust, London, UK

**Keywords:** metastases, spinal cord compression, oncology

## Abstract

**Study Design:**

A retrospective analysis of oncologist-provided prognoses vs actual survival outcomes of patients referred with Metastatic spinal cord compression (MSCC) to a supra-regional multidisciplinary team (MDT).

**Objectives:**

Prognostic scoring systems, such as the revised Tokuhashi, are commonly used to help guide the treatment of MSCC. However, scoring systems do not accommodate for the improved outcomes of contemporary cancer therapy. Oncologist-provided prognoses play an important role in real world rapid decision making. There is a paucity of evidence assessing the accuracy of the oncologist-provided prognosis. We conducted a retrospective study to evaluate this.

**Methods:**

Data was captured between January 2015 and December 2018. Patients were split into 2 groups: Group 1 (prognosis estimated <6 months) and Group 2 (prognosis estimated >6 months). Median overall survival (mOS) and hazard ratio for death (HR) was assessed. Receiver operating characteristic (ROC) analysis was performed to assess the accuracy of the oncologist’s prognosis.

**Results:**

829 patients were included. mOS in Group 1 was 5.8 months (95% CI 4.2-7.4 m), and in Group 2 mOS was not reached. Log rank test gave a Chi^2^ of 131 (*P* < .001). Cox regression analysis revealed a HR of .30 (*P* < .001). Area under the ROC curve was 78%.

**Conclusions:**

Oncologist-provided prognosis is accurate in this cohort of unselected, consecutive MSCC patients. It reduced reliance on scoring systems that can become outdated. Given the rapid progress in cancer treatment, the oncologist’s prognostic prediction is integral in efficient and effective MSCC management to help rapidly determine surgical candidacy.

## Introduction

The incidence of spinal metastases from all malignancies is approximately 5%,^1^ rising to 10%-40% in certain malignancies such as lung, prostate and breast cancers.^2^ Patients with spinal metastases are at risk of spinal cord compression, but the true incidence of Metastatic Spinal Cord Compression (MSCC) in England and Wales is difficult to estimate.^3^ Based on published data, the true global incidence is estimated to be up to 80 cases per million population per year.^1,4^ This translates to approximately 4000 cases per year in England and Wales.

Numerous studies have shown that clinical outcomes, with respect to neurological function, are related to the promptness of diagnosis and treatment, and to the treatment modality employed.^5-7^ Decisions on whether to actively treat or not, and the choice between different modalities of therapy are based on multiple factors. These include: technical feasibility of surgery or radiotherapy,^8,9^ type of primary cancer,^3^ previous therapeutic approaches^10^ and the overall patient prognosis.^2^ Prognostic scoring systems such as the Tomita and Tokuhashi scores, have are often utilised by surgeons,^11,12^ but guidelines set out by the National Institute for Health and Care Excellence (NICE) for the management of MSCC accept that the overall quality of evidence for scoring systems is poor.^3^ Indeed, Huch et al and Ulmar et al both applied the Tomita system and did not replicate its proposed usefulness,^13,14^ and several publications^11,12,15^ suggested a revision of the Tokuhashi scoring system was needed,^16^ leading to the publication of a revised version which is now itself over 15 years old.^17^

The site of primary tumour is integral to these scoring systems and the NICE guidance also differentiates patients into 3 prognostic groups based on the site of the primary cancer, noting, for example, that patients with melanoma and lung cancer will have a survival of less than 12 months.^3^ Given the rapid development in therapeutic options available for patients with these malignancies over the last few decades and the ensuing improvement in survival, the suggested prognostic timelines are likely to be an underestimation.^18^ There is some debate surrounding the optimum prognosis cut off for offering surgery, with NICE suggesting that surgery should not be offered to patients with an estimated prognosis of less than 3 months.^3^ Both Enkaoua and Tokuhashi suggested it may be useful to further differentiate the treatment options for patients with a life expectancy of more than 6-month^11,15^ Conversely, the International Spine Oncology Consortium (ISOC) report in the Lancet Oncology suggests a prognosis of two or more months being the cut off for decisions regarding surgical intervention,^19^ though this has been criticised by the NeuroSpine Surgery Research Group (NSURG) in Australia who suggest that a 6-month cut-off would be a more reasonable figure for these patients given the cost and quality of life implications of treatment.^19,20^ The NSURG review further clarified that the exception would be vertebroplasty where the cost profile is relatively minimal and recovery is quick compared to other surgical interventions. However, for separation surgery and reconstruction surgery, a 6-month cut-off should be considered. Despite the utility that scoring systems can have in defining prognosis, it should be the prognostic estimate of the treating oncologist that reigns supreme in informing treatment. At our centre we use prognosis estimates provided by the referring oncologist. A cut-off of an overall prognosis of less than 6 months is used as a benchmark figure for a decision not to use surgery as an intervention, given the risks, complications and post-operative recovery time.

Considering the impact that the decision regarding surgical intervention can have on a patient’s functional status and the value given to the estimated prognosis in making such decisions, we believe a large-scale review of the performance of such estimates is warranted. Therefore, we undertook an analysis of data over 4 years at a supra-regional quaternary specialist spinal centre performing a large number of spinal operations to evaluate the accuracy of the referring oncologist’s prognostic estimate.

## Materials and Methods

This study was registered with the St. George’s Hospital Clinical Audit and Effectiveness Department under ‘Cancer research for service evaluation’, audit number: AUDI003026. The retrospective and non-interventional nature of this study meant that patient consent was not explicitly sought.

All data for referrals to our supra-regional centre are prospectively collected and an electronic database maintained by the Spinal Multidisciplinary Team (MDT). From 2015 onwards, our centre required referrals to include an estimated prognosis by the referring clinician. However, this field has not been consistently completed. The prognosis would be a binary one - greater than or less than 6 months. Options were available for further elucidation such as >1 year or <3 months. The present study has analysed data based on a 6-month prognostic cut-off, as this is what is used to inform whether there is likely to be a net benefit of performing an invasive operation and dealing with the recovery and possible complications of such an operation given the possibly short survival time. We analysed the hospital electronic patient records, patient notes and the spinal MDT electronic database to extract data for patients referred to the MDT between the dates of January 2015 and December 2018. Final data analysis occurred in May 2019. Patients for whom an estimated prognosis was not provided (coded as unknown) were excluded from data analysis. Patients under the age of 18, and those for whom accurate follow up data was not available were also excluded from data analysis. Co-variables collected for analysis include age, sex, cancer type, presenting symptom, treatment modality, length of time to treatment, pain score, neurological symptoms and continence, performance status, mortality data and last follow up, Charlson co-morbidity index and the Index of Multiple Deprivation (IMD) for assessment of socio-economic deprivation. The IMD is the official measure of deprivation in England using 7 indices. These indices are income, employment, education, skills and training, health and disability, crime, and housing. The Charlson co-morbidity index is a weighted score to predict risk of mortality based on scores given for 22 medical conditions. This data was extracted electronically from our hospital database which is linked to the national administrative database for England and Wales, the Hospital Episodes Statistic (HES) database.

Survival times were calculated from the date of referral to our MDT until the date of death. The date of scan report was not used as there would often be a lag between the report and the referral when an estimated prognosis was provided. There were also cases for which scans were not reported before referral to our centre. Descriptive analyses, and survival outcomes were analysed using the statistical package IBM SPSS Version 25. Descriptive analyses were reported as medians, and categorical variables were reported as percentages and frequencies. Overall survival was analysed using the Kaplan Meier method with Mantel-Cox Log rank test, and hazard ratios for death were calculated using Cox Regression analysis. A two-sided *P*-value of less than .05, in all tests, was considered statistically significant. To assess the performance of the oncologist’s prognosis as a test on its own we used receiver operating characteristic (ROC) curve analysis.

## Results

A total of 1572 case notes were reviewed. After initial analysis, 829 patients were included in the study, of whom 46% (n = 383) received radiotherapy, 28% (n = 229) had surgical intervention, and 26% (n = 217) had treatment defined as ‘palliative care’ which included first line, or change in, systemic therapy. The rates of surgery in this unit are similar to other published data internationally.^14^

The presenting clinical problem was pain in the majority of patients at 52% (n = 431), weakness in 24% (n = 199), and ‘other’ in 24% (n = 199). 260 patients were given an estimated prognosis of less than 6 months, and 569 patients were given an estimated prognosis of greater than 6 months (Table 1).

**Table 1.** Patient Characteristics.

	Estimated Prognosis <6 m (n = 260)	Estimated Prognosis >6 m (n = 569)
Age		
Median (range)	73 (34-97)	68 (31-96)
IMD deprivation score		
1 (most deprived)	75 (29%)	156 (27%)
2	60 (23%)	148 (27%)
3	52 (20%)	57 (10%)
4	47 (18%)	116 (20%)
5 (least deprived)	20 (8%)	75 (13%)
Not coded	6 (2%)	17 (3%)
Charlson score		
0-2 (few co-morbidities)	39 (15%)	113 (20%)
3-5	18 (7%)	40 (7%)
6-8	94 (36%)	204 (36%)
9-12 (more co-morbidities)	31 (12%)	40 (7%)
Not coded	78 (30%)	170 (30%)
ECOG performance status		
0	116 (14%)	74 (13%)
1	34 (4%)	69 (12%)
2	265 (32%)	222 (39%)
3	198 (24%)	74 (13%)
4	0	6 (1%)
9 (not specified)	216 (26%)	124 (22%)
Continent	224 (86%)	455 (80%)
Incontinent/catheterised	16 (8%)	30 (5%)
Unrecorded	20 (7%)	27 (5%)
Pain score 0-4/10	73 (28%)	188 (33%)
Pain score 5-6/10	99 (38%)	205 (36%)
Pain score 7-10/10	88 (34%)	159 (28%)
Surgery	39 (15%)	190 (33%)
Radiotherapy	70 (27%)	313 (55%)
Palliative care (including systemic therapy)	151 (58%)	66 (12%)

## Statistical Analysis

**Figure 1.**
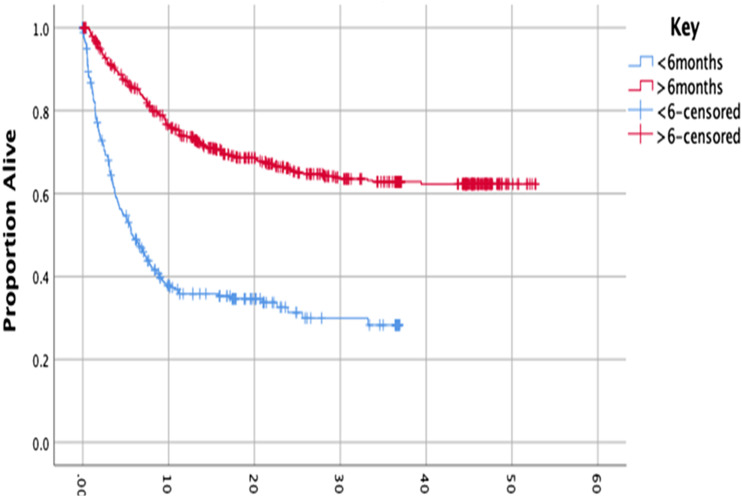
Kaplan-Meier curve analysing survival time from reporting of MRI scan, comparing the <6 m group with >6 m group.

Kaplan Meir survival analysis revealed a median overall survival (mOS) in the patients given a prognosis of <6 months was 5.8 months (95% CI 4.2-7.4 m), and in Group 2 median survival was not reached at time of data analysis (NR) (Figure 1). Log rank test gave a Chi^2^ of 131 (*P* < .001).

**Figure 2.**
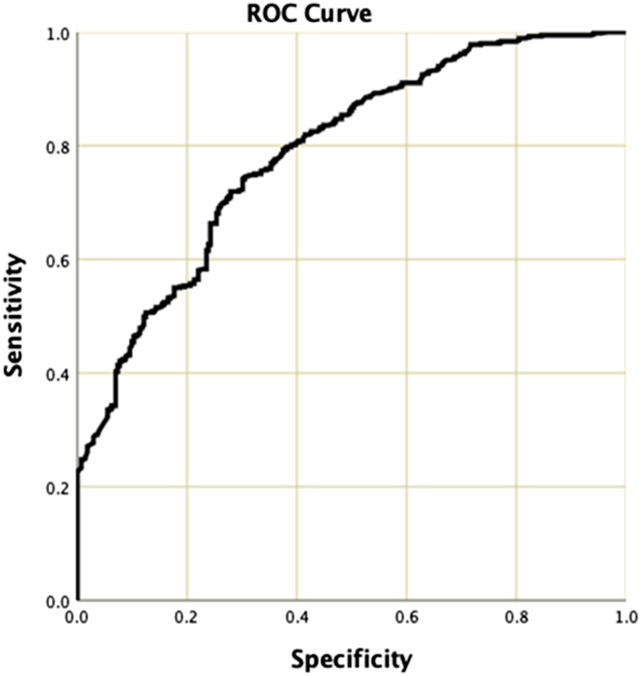
Receiver-operator curve (ROC) showing the relationship between the actual survival and the binary oncologist prognosis of <6 m or >6 m.

Cox regression analysis revealed a hazard ratio for death of .30 (*P* < .001), confirming a 70% increased risk of death over time for the <6 m estimated prognosis group. To assess the performance of the oncologist’s prognosis as a test on its own, in predicting a prognosis of less than or greater than 6 months we used receiver-operating characteristic (ROC) curve analysis. The ROC curve has an area under the curve of 78.1% (Figure 2).

In total there were 476 deaths (57.4%) during the study period. In the poor prognosis group 169 patients died within 6 months (65%), and in the good prognosis group 83 patients died within 6 months (14.5%).

## Discussion

MSCC is the second most common neurological complication of cancer following brain metastases.^21^ It has a major impact on the quality of life of patients, with features such as intractable pain, incontinence and paralysis.^22,23^ The optimal treatment of MSCC is determined by the MDT, who use the patient’s estimated prognosis as a key tool in this assessment. Traditionally, surgeons used scoring systems to estimate prognosis and guide treatment. Advances in systemic therapy can alter traditional scoring system accuracy. For example, patients given a poor prognosis may have an unusually good response to systemic therapy and exceed their estimate. In our cohort of patients, 91 of the ‘poor prognosis’ patients (35%) survived longer than six months, and 83 of our ‘good prognosis group’ (14%) died within 6 months. Patient prognosis was categorised as more than or less than 6 months to balance the impact of surgical recovery and systemic treatment side-effects with the additional quality of life provided by treatment. Whilst patient factors ultimately dictate treatment, the use of a 6-month time frame as a cut-off for surgical intervention is also useful when considering the health economics associated with the expensive surgical treatment of a patient with a poor prognosis.

Scoring systems have historically been used for prognostication in patients with MSCC.^24^ A popular,^25^ scoring system first proposed by Tokuhashi et al in 1990 and then revised in 2005, considers multiple variables for facilitating the decision of whether to operate.^11,26^ The Tokuhashi score is calculated using: the Karnofsky performance status, number of vertebral metastases, metastases to internal organs, number of extra-spinal bone metastases, primary site of cancer, and the presence of spinal cord deficit. It does not consider bony instability or acuteness of neurologic presentation. Consideration of these features provides, to some extent, a surrogate for the oncologist’s estimated prognosis. However unlike an oncologist, scoring systems fail to provide a prognostic estimate that is dynamic and capable of adapting to the latest cancer therapies, peer-reviewed literature and patient-specific knowledge.

Scoring systems are trapped in time at the point of their most recent iteration and their prognostic accuracy is limited accordingly. The improvement in cancer survival times is well documented.^27-35^ For example, in non-small cell lung cancer 5-year survival rates have improve from 10.7% in 1973 to 19.8% by 2015,^36^ in turn meaning the 1-year survival following MSCC is also improving.^37^ This improvement in prognosis is not necessarily reflected, meaning that preoperative scoring systems may lead to an artificially poorer estimated prognosis. This treatment-limiting, and thus life-limiting, underestimate is strong evidence for the need for real-life, patient-specific contextualisation when implementing these scoring systems and is why the adaptable prognostic estimate of the referring oncologist may be superior.

A further limitation of scoring systems is that they fail to account for the specific targeted therapy options that are now available for the treatment of specific malignancies with specific cancer biology. For example, the use of small molecule inhibitors in the treatment of epidermal growth factor receptor (EGFR) mutation positive lung cancers has lead to improved survival rates.^38,39^ The improved prognosis conferred by the advances in cancer-specific treatment is something that generalised scoring systems fail to account for. Accordingly, the robustness of the Tokuhashi score in the era of improved systemic therapy options for patients with MSCC secondary to lung cancer has been previously evaluated and shown to be suboptimal by Hessler, et al.^40^ Similarly, Gregory, et al have called for more stringent validation of prognostic scoring systems for metastatic spinal disease in the era of anti-vascular endothelial growth factor (VEGF) therapies.^41^ Whilst cancer-specific scoring systems have been devised with a view to overcome the limitations and inaccuracies of their more generalised counterparts, see,^42,43^ the need to design and critically evaluate multiple cancer-, and cancer-subtype, specific scoring systems for the myriad cancers that may cause MSCC as well as to continually update these scoring systems in light of advances in treatment modalities is disadvantageous^44^; certainly as compared to the dynamic and accurate prognostication of oncologists. Overall, the predictive accuracy and clinical relevance of the many scoring systems that have arisen since Tokuhashi’s first iteration in 1990 have been evaluated by several studies, but the results have been inconsistent.^9,45,46^ Whilst scoring systems are still useful adjuncts in considering MSCC, their lack of appreciation for advances in systematic cancer therapy significantly limits their utility.^47^

The neurological, oncological, mechanical and systemic (NOMS) treatment framework devised by Memorial Sloan-Kettering Cancer Centre takes a more algorithmic, multidisciplinary team approach to MSCC.^48^ A multidisciplinary team evaluates patients with respect to 4 pillars - neurological status, oncological status, mechanical instability, and systemic disease assessment. A key distinction between this system and the Tokuhashi scoring system is the dynamic oncological assessment that is integral to NOMS. An oncologist will predict the response of the tumour to current available therapies and this evaluation will underpin the overall treatment trajectory. Thus, surgery can be avoided in all patients bar those who have radioresistant tumours with high grade epidural spinal cord compression or mechanical instability.^48^ The Tokuhashi score’s comparatively discrete categorical assessment of the cancer inherently lacks this level of granularity and will therefore be less useful when it comes to treatment decisions than the NOMS framework, for which the oncologist’s input is key.^49^

Although consensus exists on the decision not to operate when prognosis is poor, there is a paucity of data evaluating the accuracy of prognostication by the oncologists. To our knowledge, only one study, comprising just 55 patients, has made direct comparison between the accuracy of oncologist-provided prognoses and that of scoring systems (revised Tokuhashi); with the oncologists being significantly more accurate.^50^ Our own data also demonstrates oncologists to be accurate, supporting their findings. This, in addition to studies which have identified the revised Tokuhashi score as having an unacceptably low accuracy,^19,50,51^ further highlights the need for oncologists’ opinions to be weightier than objective scores when it comes to prognostication.

The two main reasons for exclusion from the study were: no prognosis given, and patients referred who did not have malignant spinal disease. Other reasons included not having any follow up data - this is a particular problem in supra-regional centres where referrals are made from far afield, and in some cases it was unclear if patients still resided within England. This is a retrospective, single quaternary centre analysis and is limited accordingly, for example missing data inputs and unnoticed biases may have impacted the quality of this study.^52^ It may also be argued that the prognosis given at the point of deciding whether or not to treat MSCC is self-perpetuating, as being given a prognosis of <6 months itself may influence survival by way of the impact it may have on subsequent treatment decisions. These limitations suggest caution in drawing definitive conclusions from our results. However, such potential confounders are impossible to account for robustly, as subsequent treatment decisions are themselves multifactorial, with inter-clinician variability. Ultimately these factors are unlikely to be entirely responsible for the differences seen. Our results confirm that the prognosis given by oncologists as part of the multidisciplinary decision is accurate for this large cohort of unselected, consecutive ‘real world’ patients.

In the era of contemporary cancer treatment the role of machine learning and artificial intelligence cannot be understated. The development of machine learning algorithms such as that devised by the Skeletal Oncological Research Group (SORG) has been shown to effectively predict 90-day and 1 year mortality in patients with spinal metastatic disease.^53,54^ The development of such algorithms certainly has synergistic value with the accurate prognoses provided by oncologists and adds to the roster of tools at the disposal of oncologists to correctly predict prognoses and inform the treatment of patients with MSCC in the MDT setting.

## Conclusion

This study supports the view that an oncologist’s prediction of survivorship is an accurate and crucial component of the Spinal Oncology MSCC MDT decision making process. It adds to the mounting evidence that, as cancer treatment continues to advance and scorings systems become increasingly inaccurate, the patient-specific opinion of the oncologist remains an integral part of surgical decision making. Ideally this assessment would be part of a wider NOMS framework to better appreciate the multifaceted nature of MSCC. In conclusion, the referring oncologists to our quaternary MSCC service are able to give accurate predictions of life expectancy which greatly assists a rapid and robust MDT response to aid in the decision making of patients with MSCC.
